# Stromal SLIT2 impacts on pancreatic cancer-associated neural remodeling

**DOI:** 10.1038/cddis.2014.557

**Published:** 2015-01-15

**Authors:** V Secq, J Leca, C Bressy, F Guillaumond, P Skrobuk, J Nigri, S Lac, M-N Lavaut, T-t Bui, A K Thakur, N Callizot, R Steinschneider, P Berthezene, N Dusetti, M Ouaissi, V Moutardier, E Calvo, C Bousquet, S Garcia, G Bidaut, S Vasseur, J L Iovanna, R Tomasini

**Affiliations:** 1 CRCM, Cellular Stress, INSERM, U1068, Parc scientifique de Luminy, Paoli-Calmettes Institute, Aix-Marseille University, UM 105, CNRS, UMR7258, Marseille 13009, France; 2 Department of Pathology, Hospital North/Mediterranean University, Marseille, France; 3 Neuronexperts, Medical North Faculty, Marseille, France; 4 Aix-Marseille University, INSERM, CRO2, UMR 911, Marseille 13385, France; 5 Molecular Endocrinology and Oncology Research Center, CHUL Research Center, Quebec City, QCue, Canada; 6 INSERM UMR 1037, CRCT, University Toulouse III, Toulouse, France

## Abstract

Pancreatic ductal adenocarcinoma (PDA) is a critical health issue in the field of cancer, with few therapeutic options. Evidence supports an implication of the intratumoral microenvironment (stroma) on PDA progression. However, its contribution to the role of neuroplastic changes within the pathophysiology and clinical course of PDA, through tumor recurrence and neuropathic pain, remains unknown, neglecting a putative, therapeutic window. Here, we report that the intratumoral microenvironment is a mediator of PDA-associated neural remodeling (PANR), and we highlight factors such as ‘SLIT2' (an axon guidance molecule), which is expressed by cancer-associated fibroblasts (CAFs), that impact on neuroplastic changes in human PDA. We showed that ‘CAF-secreted SLIT2' increases neurite outgrowth from dorsal root ganglia neurons as well as from Schwann cell migration/proliferation by modulating N-cadherin/*β*-catenin signaling. Importantly, SLIT2/ROBO signaling inhibition disrupts this stromal/neural connection. Finally, we revealed that SLIT2 expression and CAFs are correlated with neural remodeling within human and mouse PDA. All together, our data demonstrate the implication of CAFs, through the secretion of axon guidance molecule, in PANR. Furthermore, it provides rationale to investigate the disruption of the stromal/neural compartment connection with SLIT2/ROBO inhibitors for the treatment of pancreatic cancer recurrence and pain.

Even after significant efforts from the scientific community in the past decade, pancreatic ductal adenocarcinoma (PDA) remains one of the most lethal cancers with worrying predictions.^1^ Median survival stagnates around 5 months, together with a 5-year survival at 5%. For 5–20% of patients treated surgically, the 5-year survival reaches 20%, with a median survival of 16 months. Metastasis onset and high prevalence of local tumor recurrence after potential curative resection influence patient's survival. A recent study revealed that the overall survival of patients with tumor recurrence was 9.3, *versus* 26.3 months for patients without early relapse.^2, 3^ Although several causes are attributed to local recurrence, reports highlight intrapancreatic nerve invasion as a predictor for recurrence^4^ by playing the role of a specific niche for scattered tumoral cells. In light of such epidemiologic data, there is a crucial need to develop optimal therapeutic strategies, taking into account the tumoral cellular composition, over the next decade.^5^

Tumors are complex tissues in which mutant cancer cells and subverted normal cells coexist and interact to form an intricate network. This is even more accurate for PDA, in which tumor stroma (intratumoral microenvironment), representing 90% of the tumor mass, is considered as an emerging hallmark.^6^ It is composed of the extracellular matrix, mesenchymal cells (cancer-associated fibroblasts, CAFs), blood and lymphatic vessels, nerve fibers and inflammatory cells. This stromal compartment is particularly active in tumor development^7, 8^ and clinical outcome.^9^ However, although genetic changes in tumor epithelial cells have been deeply investigated in the past decades,^10^ investigation into the role of stromal cells has largely lagged behind and is nowadays of major interest.

Beyond the presence of an important stromal compartment, another characteristic of PDA is the presence of a modified innervation in nearly all patients. This includes increased neural density, hypertrophy and pancreatic neuritis, as well as intra- and extrapancreatic perineural invasion (PNI) by cancer cells.^11, 12^ This neural remodeling or PDA-associated neural remodeling (PANR) is clinically correlated with neuropathic pain^12, 13^ and locoregional spread^14^ and is a marker of poor prognosis.^15, 16^ As noted above, several studies reported neural remodeling as one reason for local tumor recurrence after curative resection, with residual tumor cells present in the remnant pancreas nerves.^17^ Moreover, PNI was recently shown as an independent prognostic factor^18^ and even as the more accurate predictor for recurrence.^19^ Beyond a clear clinical significance, PANR pathogenesis and associated molecular mechanisms are still poorly understood.^20, 21, 22, 23^ Improvement of our knowledge on molecular pathways underlying PANR may lead to better prognostic indicators as well as to innovative therapeutic strategies targeting local recurrence and locoregional spread, as well as cancer-associated pain.

Here, we realized microarray transcriptomic analysis of stromal *versus* tumoral cell compartments from several PDA patients, and highlighted stromal neurogenic factors potentially impacting on PANR. Among them, we further examined the specific involvement of axon guidance molecules SLIT2 within *in vitro* and *in vivo* models. Finally, our results reveal a key role of intratumoral microenvironment on PANR and suggest that inhibiting tumor–stroma interactions could be a promising therapeutic strategy to hold down processes involved in disease recurrence and associated neuropathic pain.

## Results

### Determination of stromal and tumoral cell compartment transcriptomic signatures and characterization of an enriched ‘axon guidance' family

Clinical hallmarks of PDA are the abundant stromal reaction and the presence of PANR (Supplementary Figure 1A), which have been widely separately documented.^11^ However, the connection between these two processes is limited to a unique study revealing that neuronal plasticity can be induced by extracts from PDA's stromal compartment.^24^ To analyze the impact of the intratumoral microenvironment on PANR, we decided to decipher the transcriptomic profile of the stromal cell compartment within the human PDA tissue. We used laser capture microdissection on human PDA samples to separate epithelial cells from stromal ones and analyzed their relative gene expression profiles using the Affymetrix U133 gene chip set followed by significance analysis of microarray (SAM) analyses (Supplementary Figures 1B and C; GEO repository GSE50570). We then realized a GO global enrichment analysis using only genes overexpressed in the stromal compartment with a significant *P*-value (1001 genes) and highlighted an ‘axon guidance' family including 48 genes with an enrichment *P*-value of 5.42E^−8^ (Supplementary Figure 1D). This family represents a total of 4.8% of all genes overexpressed in the stromal compartment within PDA. Those data indicate that the stromal compartment within PDA specifically produces molecules that could impact on axon guidance and nerve cell abilities, and thus on PANR.

### Among the key factors from the intratumoral microenvironment, SLIT2, an axon guidance molecule, is mainly expressed by CAFs

**Figure 1:**
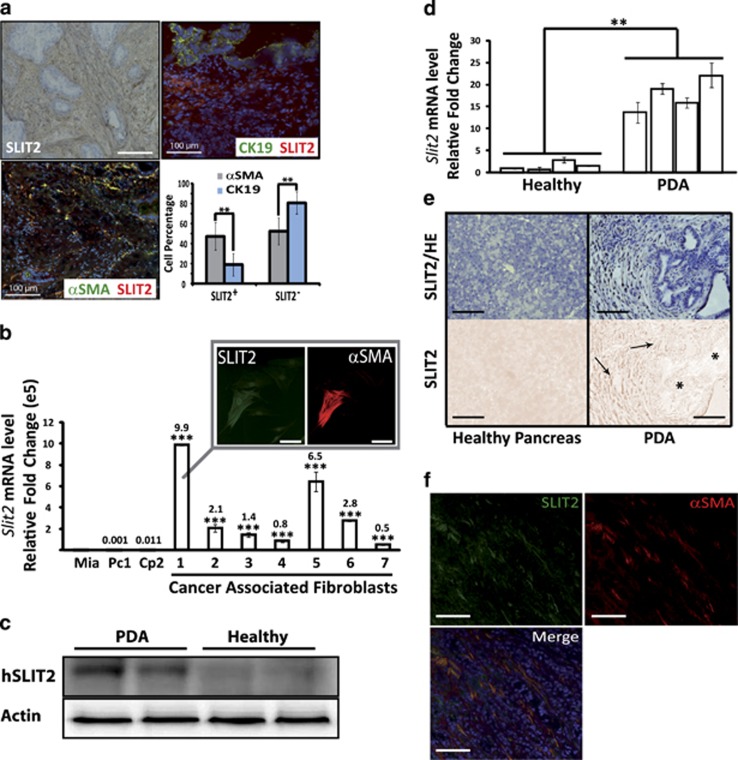
SLIT2 expression pattern in CAFs of the stromal compartment within human (h) and mouse PDA. (**a**) IHC and IF images of human PDA stained for SLIT2 (upper left), SLIT2 (red) and cytokeratin 19 (CK19) (green) in the upper right and SLIT2 (red) and *α*SMA (green) in the lower left. Fluorescence intensity quantification (of IF staining) was carried out on four human PDA (magnification: × 20) (**P<0.01) using Image J software. (**b**) SLIT2 mRNA quantification by quantitative real-time PCR (QRT-PCR) in CAFs extracted from seven different human PDA *versus* human pancreatic tumoral cell lines, Miapaca2 (Mia) as reference (value at 0.00001e^5^), Panc1 (Pc1) and Capan-2 (Cp2) (***P<0.001). (**b**, inset) IF representative images of CAFs stained for SLIT2 (green) and *α*SMA (red) (magnification: × 20). (**c**) Western blot of SLIT2 expression in protein extracts from human PDA or healthy human pancreas (demonstrating the increased expression of SLIT2 in PDA extracts). Actin was used as a loading control. (**d**) SLIT2 mRNA quantification by QRT-PCR in the pancreas of 8-week-old healthy mice (Kras^G12D^/Ink4A^f/f^) and PDA-bearing mice (pdx1-cre/Kras^G12D^/Ink4A^f/f^) (**P<0.01). (**e**) SLIT2 expression and HE counterstaining of pancreas from 8-week-old healthy mice and PDA-bearing mice (upper images) and SLIT2 staining (lower images). *, Epithelial/ductal tumoral cells/structures; arrows, stromal/fibroblast compartment. (**f**) Representative IF images of CAFs labeled with *α*SMA (red) and SLIT2 (green) in mouse PDA (magnification: × 20; scale bar: 100 *μ*m)

As PANR is characterized by an increased nerve density, this ‘axon guidance' family seems conceptually relevant. Indeed, such a process could be involved in the attraction and growth of new and/or existing nerve fibers within PDA tumor. Among those 48 genes present in the ‘axon guidance' family (Supplementary Figure 1D), we observed two members (ROBO1 and SLIT2) of a well-known axon guidance axis, the SLIT/ROBO signaling pathway, recently associated with pancreatic cancer genome aberration and patient survival.^10^ Moreover, *SLIT2* is one of the most overexpressed genes among ‘axon guidance' genes, with a 13.9-fold increased expression (Supplementary Figure 1D). We first analyzed *in vivo* the expression pattern of SLIT2 and showed by immunohistochemistry (IHC) and immunofluorescence (IF) (Figure 1a) that it was mainly expressed in the stromal compartment and more precisely within CAFs, as shown by coimmunostaining with *α*-smooth muscle actin (*α*SMA), a PDA CAF marker. SLIT2 expression in CAFs was also shown using primary CAFs extracted from seven different patients, and a higher expression than in epithelial tumoral cells was confirmed with a fold increase range of 4.9e4–9.9e5 depending on primary CAF culture (*P*<0.001; Figure 1b). More importantly, we show that SLIT2 level is increased in PDA compared with healthy pancreas in human (Figure 1c) as well as in an endogenous mouse model: the pdx1-cre/Kras^G12D^/Ink4A^f/f^ mice^25^ (Figure 1d). In this mouse model, Slit2 was also localized in the stromal compartment by IHC (Figure 1e) and more specifically in CAFs (Figure 1f). Altogether, our *in vivo* and *in vitro* data suggest that the stromal compartment and, more precisely, CAFs express the axon guidance molecule, SLIT2, which could impact on PANR.

### *In vitro* modeling of intratumoral microenvironment cell interactions

**Figure 2:**
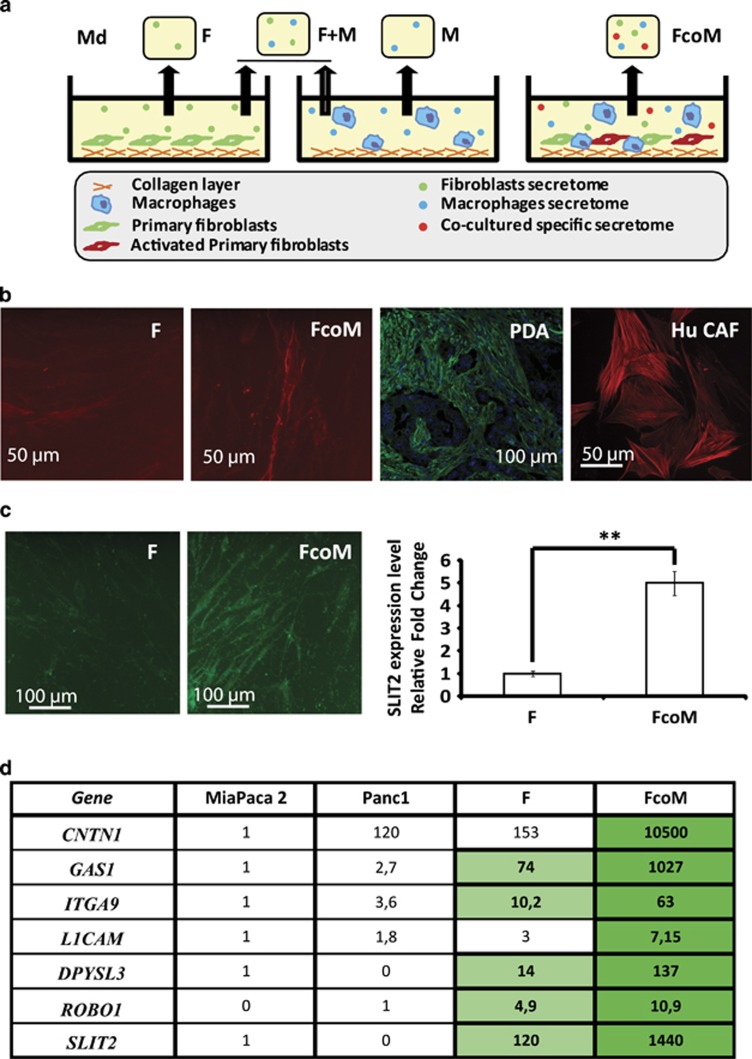
*In vitro* model and axon guidance molecule expression *in vitro*. (**a**) Graphical representation of the technical procedure used to mimic *in vitro* the PDA intratumoral compartment and to produce conditioned media (Cd media). (**b**) IF images of *α*SMA staining on human fibroblasts alone (F) or cocultured with macrophages (FcoM), human PDA (PDA) or CAF from human PDA (magnification: × 20 and × 40 for *in vivo* and *in vitro* images, respectively). (**c**) Representative images of fibroblasts (F) or fibroblasts cocultured with macrophages (FcoM) labeled for SLIT2 (green). SLIT2 quantification from IF staining on fibroblasts (F, used as normalizer) or fibroblasts cocultured with macrophages (FcoM) (*n*=3; **P<0.01). (**d**) Human mRNA level of some genes composing the ‘axon guidance' family was analyzed by quantitative real-time PCR (QRT-PCR) in human pancreatic tumoral cell lines (Miapaca2 used as normalizer, except for *ROBO1,* which are not expressed in Miapaca2, and Panc1), human fibroblasts (F) and human fibroblasts cocultured with mouse macrophages (FcoM). Light green, *P*<0.05 *versus* Panc1; dark green, *P*<0.05 *versus* F (*n*=3). Md, conditioned media from: SNF, sNF 96.2; F, fibroblasts; M, macrophages; F+M, mixed Md from separated cultures of fibroblasts and macrophages; FcoM, cocultures of fibroblasts and macrophages

To analyze the impact of the stromal compartment on PANR *in vitro*, we designed an *in vitro* model constituted of human primary fibroblasts and murine macrophages cultivated on collagen matrix (Figure 2a). We first checked the relevance of this model and observed that primary fibroblasts cocultivated with macrophages show increased expression of *α*SMA, a well-known CAF marker within PDA (Figure 2b). This suggests that, in coculture condition with macrophages, autocrine/paracrine components are able to switch on the activation process of primary fibroblasts by turning them into CAF-like cells mimicking the intratumoral microenvironment. Interestingly, in those culture conditions, the protein level of SLIT2 is increased 5.1±0.3-fold in fibroblasts cultivated alone and in fibroblasts cocultured with macrophages (*P*<0.01; Figure 2c). To validate such a concept with our own data, we analyzed the expression level of several members of the ‘axon guidance' family highlighted by gene enrichment, and observed a CAF-like expression pattern, as all members tested were overexpressed in fibroblasts cocultured with macrophages, compared either with fibroblasts alone or with epithelial tumoral cells (Figure 2d), whereas none of them were increased in macrophages cocultured with fibroblasts (Supplementary Figure 2). These data suggest that our *in vitro* model can mimic the PDA intratumoral microenvironment and thus could be used as an efficient *in vitro* model to decipher the modulation and impact of the intratumoral microenvironment on PANR.

### Media from our *in vitro* model induce changes in neuron and Schwann cell behaviors

**Figure 3:**
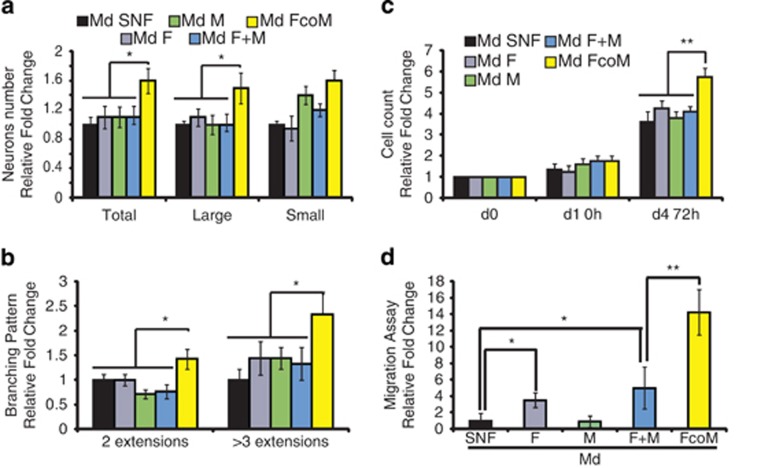
Intratumoral microenvironment induces changes in the primary sensory neurons (**a** and **b**) and Schwann cell behaviors (**c** and **d**). (**a**) Growth in primary sensory neurons (Total, large and small neurons) cultured for 5 days in various conditioned media (Md) (*N*=3). (**b**) Branching pattern was measured in 5-day cultured sensory neurons by counting the number of neuritis/extensions by neurons (*N*=3). (**c**) Human Schwann cell proliferation was measured by cell counting 72 h after incubation with various conditioned media. (**d**) Migration assay: human Schwann cells were assessed for migration abilities on Boyden chamber assay for 4 h (*n*=3; *P<0.05; **P<0.01). Md, conditioned media from: SNF, sNF 96.2; F, fibroblasts; M, macrophages; F+M, mixed Md from separated cultures of fibroblasts and macrophages; FcoM, cocultures of fibroblasts and macrophages

So far, a unique study has revealed that pancreatic tumor extracts (mix of proteins from every cell type composing PDA) can induce neuronal plasticity through the increase of neurite density and neuronal branch length.^24^ To definitively confirm the strength of our *in vitro* model and also emphasize our leading hypothesis on the role of the stromal compartment on PANR, we first submitted dorsal root ganglion (DRG) neurons to our stromal conditioned media (which showed the overexpression of 9/15 genes from the ‘neurogenic factor family') *versus* control conditioned media. Interestingly, we observed that stromal conditioned media (FcoM) significantly increase the total number of neurons (relative fold change compared with control: 1.6±0.17, *P*<0.05) and, more specifically, large neurons (relative fold change compared with control of 1.5±0.21, *P*<0.05; Figure 3a), and favor neuronal networks (Supplementary Figure 3A) and branching pattern (relative fold change compared with control of 1.4±0.15 for two extensions, *P*<0.05, and 2.3±0.3 for three or more extensions, *P*<0.05; Figure 3b), which represent crucial parameters for nerve formation, extension and regeneration after an injury or within physiopathological circumstances.^26^ These data, which validate previous observations,^24^ further highlight the direct implication of the intratumoral microenvironment in PANR.

However, while neuronal modulation is important for neural remodeling, processes associated with Schwann cell behavior (main nerve fiber cell components tightly associated with neurons) are also involved in fiber attraction and sprouting.^27^ Interestingly, we showed that stromal conditioned media (FcoM) significantly enhance Schwann cell proliferation (5.75±0.4 *versus* 3.6±0.3 for control condition) (cell counting fold change; *P*<0.01 (Figure 3c) and mitochondrial activity; *P*<0.05 (Supplementary Figure 3B)) as well as Schwann cell migration, with 14.3±2.7 for FcoM media *versus* 5±2.5 for F+M media and 1±0.9 for SNF media (*P*<0.05 and *P*<0.01; Figure 3d). All together, our data confirmed that the intratumoral microenvironment is able to modify several neurons and Schwann cell abilities that can be related to processes involved in PANR.

### The SLIT2/ROBO pathway impacts directly on nerve cell behaviors linked to PANR

**Figure 4:**
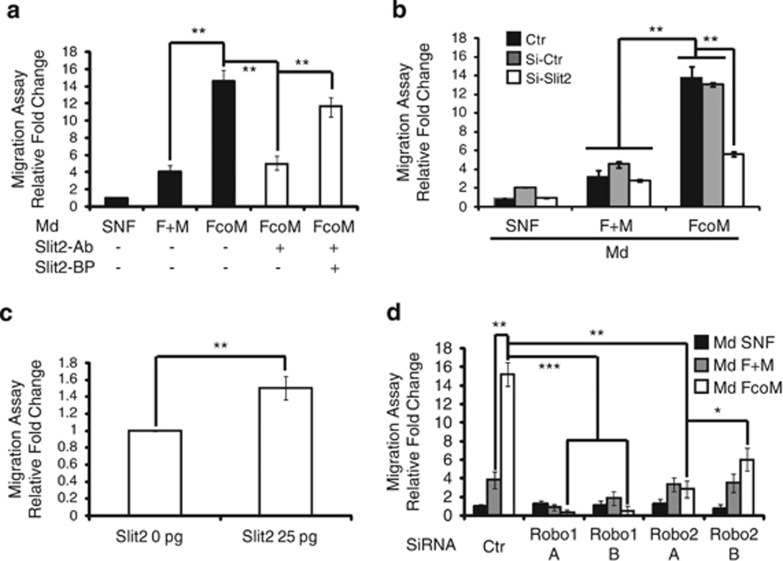
SLIT2/ROBO pathway impacts on neural cells behaviors linked to PANR. (**a**–**d**) Migration assay: human Schwann cells were assessed for migration abilities on Boyden chamber assay for 4 h with (**a**) various conditioned media±SLIT2 antibody (Slit2-Ab) to deplete SLIT2 from the conditioned media±blocking peptide (SLIT2-BP) as control. (**b**) Conditioned media from control (SNF), mixed (F+M) or cocultures (FcoM) using fibroblasts transfected with control or SLIT2 targeting siRNA. (**c**) SNF conditioned medium supplemented, or not, with 25 pg of human recombinant SLIT2. (**d**) Conditioned medium from control (SNF), mixed (F+M) or cocultures (FcoM) applied on Schwann cells transfected with control or Robo1 (A and B) or Robo2 (A and B) targeting siRNA. (**a**–**d**) (*n*=3; *P<0.05; **P<0.01; ***P<0.001). Md, conditioned medium from: SNF, sNF 96.2; F, fibroblasts; M, macrophages; F+M, mixed Md from separated cultures of fibroblasts and macrophages; FcoM, cocultures of fibroblasts and macrophages

To determine the real impact of SLIT2 and consequent signaling on PANR, we took advantage of our *in vitro* model. Following our hypothesis that CAFs produce SLIT2, which then impacts on PANR, we first depleted secreted SLIT2 in the conditioned medium. As suspected, we observed that the increase in Schwann cell migration induced by fibroblasts cocultured with macrophages in the conditioned medium (14.2 *versus* 4.2 for FcoM media, *P*<0.01) is lost after SLIT2 depletion (14.2–4.3, *P*<0.01; Figure 4a). This result is specifically due to SLIT2 depletion, as adding a blocking peptide to the medium reverts the phenotype (4.3–11.9, *P*<0.01; Figure 4a). This suggests that SLIT2 within the FcoM medium seems to be responsible for the enhanced Schwann cell migration. To confirm this hypothesis, we silenced SLIT2 mRNA in fibroblasts using siRNA (Supplementary Figure 4A). We showed that the conditioned medium from SLIT2-deficient fibroblasts cocultured with macrophages no longer increases Schwann cell migration (15.6-fold for FcoM Si-Ctr to 5.9-fold for FcoM Si-Slit2, *P*<0.01; Figure 4b). In this context, we also show that SLIT2 recombinant protein enhances Schwann cell migration by 1.5±0.1-fold (*P*<0.01; Figure 4c). These data confirm that SLIT2, produced by fibroblasts when cocultured/activated with macrophages, is able to improve Schwann cell migration abilities.

The SLIT2 ligand is recognized and binds to members of the ROBO receptor family.^28^ According to our model, Schwann cells used in the above experiments should express ROBO receptors, and its inhibition by siRNA should impair SLIT2 effects on migration. As shown in Supplementary Figures 4B–D, ROBO receptors are expressed in Schwann cells *in vitro* as well as in nerve fibers within human PDA samples. After validation of our siRNA targeting ROBO receptors in Schwann cells (Supplementary Figures 4C and D), we could show that ROBO1 and ROBO2 depletion in Schwann cells markedly impaired the induction of Schwann cell migration owing to the FcoM conditioned medium (*P*<0.001 for Robo1 depletion and *P*<0.05/0.01 for Robo2 depletion; Figure 4d). These data strengthen our hypothesis on the impact of the intratumoral microenvironment on PANR through the direct implication of SLIT2.

### SLIT2 modulates N-cadherin/*β*-catenin signaling to influence Schwann cells' migratory ability

**Figure 5:**
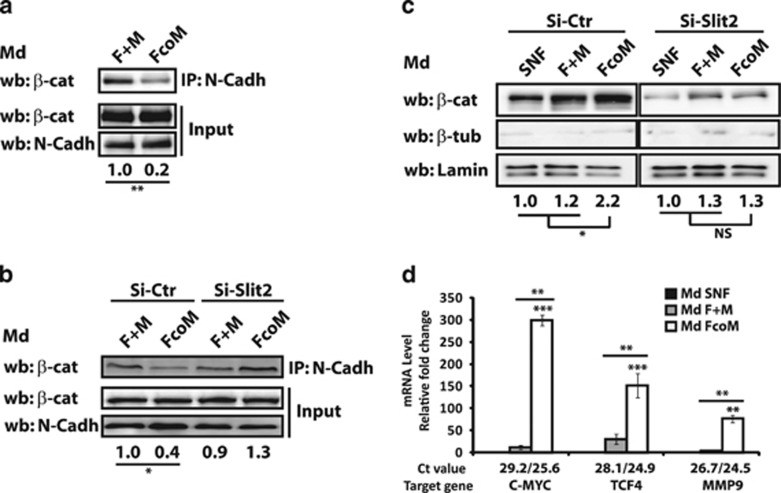
SLIT2 modulates N-cadherin (N-Cadh)/*β*-catenin (*β*-cat) signaling to influence Schwann cell migration ability. (**a**) N-cadherin/ *β*-catenin binding was analyzed by co-immunoprecipitation (IP) in SNF cells after incubation with F+M (control) or FcoM media. (*n*=3). (**b**) N-cadherin/*β*-catenin binding was analyzed by co-immunoprecipitation in SNF cells after incubation with F+M (control) or FcoM media from fibroblasts transfected with Ctr (Si-Ctr) or SLIT2 (Si-SLIT2) siRNA. (*n*=3). (**c**) Nuclear extracts from SNF cells incubated with SNF, F+M or FcoM media (with fibroblasts transfected with Si-Ctr or Si-SLIT2 siRNA) were analyzed for *β*-catenin expression. Lamin A/C was used as a loading control and *β*-tubulin was used asa quality control of nuclear extracts. *β*-Catenin expression was normalized to respective lamin A/C expression (*n*=3). (**d**) mRNA level of three *β*-catenin targets (C-MYC, LCF4 and MMP9) analyzed by quantiative real-time PCR (QRT-PCR) in SNF cells after incubation with SNF (used as normalizer), F+M or FcoM media (*n*=3; *P<0.05; **P<0.01; ***P<0.001). Md, conditioned media from: SNF, sNF 96.2; F, fibroblasts; M, macrophages; F+M, mixed Md from separated cultures of fibroblasts and macrophages; FcoM, cocultures of fibroblasts and macrophages

The highly conserved SLIT family, and their receptors ROBO, are well known to participate in central nervous system patterning as well as in sensory axon elongation and branching.^28^ Binding of SLIT2 to Robo inhibits N-cadherin-mediated adhesion by inducing the separation of *β*-catenin from the cytoplasmic part of N-cadherin leading to its direct nuclear localization where it alters the transcription of migration/proliferation targets.^29, 30^ Thus, we investigated whether SLIT2-mediated impact on Schwann cell abilities could be due to an activation of the N-cadherin/*β*-catenin pathway in these cells. We first examined N-cadherin/*β*-catenin binding and observed that FcoM medium decreases their co-immunoprecipitation after 30 min (1 for F+M media *versus* 0.2±0.1 for FcoM media, *P*<0.01; Figure 5a). We confirmed that this effect was correlated with the presence of SLIT2 within FcoM conditioned medium using conditioned medium established with fibroblasts transfected with control (Si-Ctr) or Slit2 (Si-SLIT2) siRNAs. Indeed, the use of FcoM media from si-Ctr-treated fibroblasts cocultured with macrophages showed a decrease in N-cadherin/*β*-catenin binding (1±0.2 *versus* 0.4±0.3, *P*<0.05), whereas the use of FcoM media from si-SLIT2-treated fibroblasts cocultured with macrophages did not reveal any changes (0.9±0.3 *versus* 1.3±0.3, NS; Figure 5b). We then studied the consequent translocation of free *β*-catenin into the nucleus by analyzing nuclear extracts from SNF cells incubated with various conditioned media and revealed by western blot (Figure 5c) and by IF (Supplementary Figure 5) that FcoM media increase the nuclear *β*-catenin amount (1 *versus* 2.2±0.3, *P*<0.01; Figure 5c, left panel). This effect was specifically related to the presence of SLIT2 as FcoM media from si-SLIT2-treated fibroblasts cocultured with macrophages could not induce such *β*-catenin translocation (2.2±0.3 *versus* 0.9±0.2, *P*<0.01; Figure 5c, right panel). Finally, we confirmed that nuclear *β*-catenin was transcriptionally active as FcoM medium is able to significantly increase the expression level of some of its targets (C-MYC, TCF4 and MMP9) known to be related to Schwann cell migration (Figure 5d). These results indicate that FcoM medium, through the presence of SLIT2, is able to induce the activation of the *β*-catenin pathway by having an impact on the proliferative and migratory abilities of Schwann cells.

### SLIT2 expression is correlated with PANR *in vivo*

**Figure 6:**
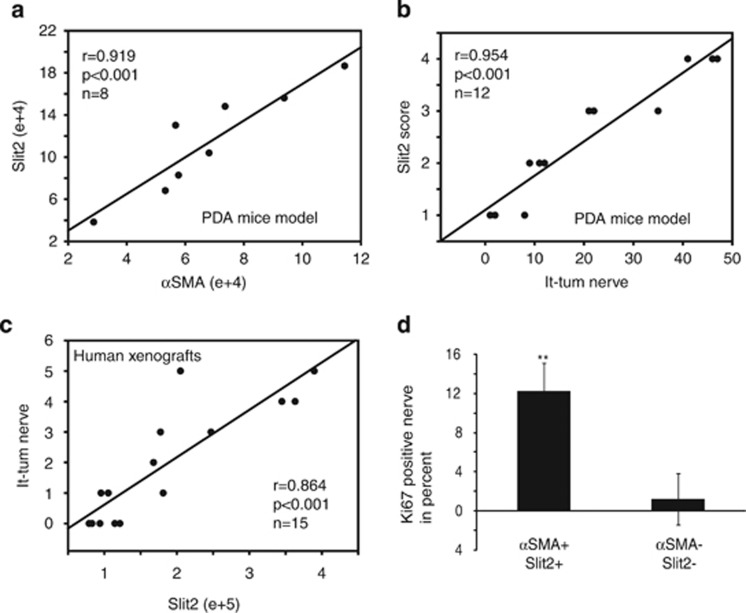
SLIT2 is positively correlated with PANR *in vivo*. (**a**) Correlation between *α*SMA expression and SLIT2 level in pancreatic tumor samples from eight PDA-bearing mice. (**b**) Correlation between Slit2 score and the number of intratumoral nerves (It-tum nerve) in pancreatic tumor samples from 12 PDA-bearing mice. (**c**) Correlation between SLIT2 level and number of intratumoral nerves (It-tum nerve) in 15 human PDA xenograft samples. (**d**) Count of nerves positive for Ki67 staining in Schwann cells, using 10 mouse PDA samples with high (*n*=5) or low (*n*=5) SLIT2 level

To reinforce the *in vivo* and *in vitro* data obtained, we investigated the correlation between Slit2 and *α*SMA expression levels *in vivo* within PDA from endogenous mouse models and found a high positive correlation between both stainings (Figure 6a and Supplementary Figure 6A). This suggests that Slit2 within PDA is dependent on the presence of CAFs. To determine whether SLIT2 level could be correlated with PANR and nerve density, we counted intra- and peritumoral nerves in PDA from 14 pdx1-cre/Kras^G12D^/Ink4A^f/f^ mice (Supplementary Figure 6A). Interestingly, we found a positive and significant correlation (r=0.954; *P*<0.001) between intratumoral nerve and SLIT2 level (Figure 6b). A positive correlation was also found between peritumoral as well as total nerve count and SLIT2 level (Supplementary Figure 6B). We decided to validate these correlations on human samples using xenograft tumors generated in nude mice by implanting pieces of freshly resected human PDA tumors. As described previously, we also revealed a positive and significant correlation between nerve count (peri, intra and total) and SLIT2 level (*P*<0.001; Figure 6c and Supplementary Figure 6C). Finally, to correlate these *in vivo* data with previously shown information of SLIT2 impact on the proliferation rate of Schwann cells, we analyzed the proliferation rate of Schwann cells *in vivo.* We revealed that nerves with Ki67-positive Schwann cells in mouse PDA tumors with a high level of SLIT2 (*α*SMA+/SLIT2+) are significantly increased compared with nerves present in mouse PDA with a low level of SLIT2 (*α*SMA−/SLIT2−) (Figure 6d). All together, these data confirm that SLIT2 produced by CAFs within the PDA stromal compartment *in vivo* is related to nerve fiber density.

## Discussion

Over the past 25 years, the vast majority of studies concentrated on the possible shortcomings of pancreatic tumoral cells and designed specific drugs/molecules targeting those cells. However, PDA is important among solid cancers because of its cellular composition. Data from an accurate mouse model of PDA revealed that the poor response of patients to systemic therapies could be due to two major concomitant morphological characteristics of these tumors: their deficient vasculature and the presence of a dense stromal matrix.^7, 8^ Indeed, several recent lines of evidence have highlighted the impact of massive hypoxic features on PDA progression through the modulation of tumor metabolism and metastasis onset,^31^ and its implication in chemotherapy resistance owing to deficiencies in blood vessels. So, tumoral architecture is a key parameter to integrate before to reach success in curative treatment.

In the past 5 years, several groups have reported that neural compartment modulation within PDA significantly influences the patient's survival and life quality.^13^ Through significant action on tumor recurrence^4, 19^ or neuropathic-associated pain,^12^ the neural compartment evolves within the intratumoral microenvironment and interacts with stromal and tumoral cells. Here, we provide evidence that the intratumoral microenvironment and, more specifically, CAFs, through production/secretion of axon guidance impacting molecules, influence the PANR by modulating various cellular processes in neurons and Schwann cells (Supplementary Figure 7), as supported by the following results: (i) the PDA stromal transcriptomic signature, obtained after microdissection of human PDA samples, revealed an ‘axon guidance'-enriched family containing 48 genes, thus 4.8% of total PDA stromal transcriptiomic signature; (ii) i*n vitro* heterotypic cultures, designed to mimic the intratumoral microenvironment cell dialog, confirmed that members of the ‘axon guidance' family were mainly expressed in the stromal compartment rather than in the tumoral epithelial cell compartment and suggested that CAFs were specifically involved; (iii) conditioned media from *in vitro* heterotypic cultures induce phenotypic changes in DRG neuron behaviors and Schwann cell abilities that could be associated with the PANR phenotype. Among key factors from the PDA intratumoral microenvironment, Slit2, an axon guidance molecule, (iv) is produced by CAFs and (v) impacts on similar neural cell abilities associated with PANR, through the modulation of the N-cadherin/*β*-catenin pathway in Schwann cells. (vi) Finally, we showed that SLIT2 level could influence PANR within endogenous mouse models of pancreatic cancer (pdx1-cre/Kras^G12D^/Ink4A^f/f^) and human PDA xenografts in nude mice, as the SLIT2 level correlated with increased intratumoral nerve fiber density.

This study reveals for the first time the PDA transcriptional signature of stromal and tumoral compartments from the same patient. Interestingly, we observed after bioinformatic analysis that the stromal signature includes mainly genes encoding for secreted factors, while the tumoral signature involves mainly genes related to cell behaviors (proliferation, apoptosis, traduction, migration, and so on). This is consistent with ongoing hypothesis and knowledge as the stromal compartment is lowly proliferative and highly secreted, while the tumoral cell compartment is highly proliferative/migrating and resistant to apoptosis.^7^ Describing CAFs as a ‘secretory machine' suggests that those cells constitute a key cell component of PDA, which is able to influence all functional dialogs involved in PDA. By direct (as mentioned in this report) or indirect action, that is, modulation of immune cells,^32^ we hypothesize that numerous genes from this stromal signature can impact on neural remodeling, among other processes.

To realize the present study, we designed *in vitro* cultures that mimic the intratumoral compartment. For this purpose, we used mesenchymal cells as well as macrophages to represent the stromal compartment. By cultivating those cells alone or in combination, we observed that fibroblasts in cocultures with macrophages shifted their activation status to become activated as CAFs would be. This suggests that macrophages have the ability to activate fibroblasts and improve the range of data showing macrophages as an important mediator of PDA progression and gemcitabine resistance.^33^ An in-depth analysis of the activation mode of those macrophages could be carried out to determine whether in our model IL-6 secretion is also responsible for macrophage changes and also to determine the phenotypic changes of these macrophages together with clinical correlation and patient's survival/status.

All data presented here support the fact that the intratumoral microenvironment, that is, CAFs through the secretion of axon guidance molecules, impacts on neural remodeling by increasing nerve density within PDA compared with healthy pancreas. However, three major questions remain: (i) How do some tumoral cells have the ability to integrate nerve structures (between the endoneurium and the perineurium mainly) to use those contaminated nerve fibers as a route for locoregional spread and consequent regional invasion or local recurrence after resection? (ii) How do those new nerve fibers acquire their pain-related activation status and how can we block the intercompartment dialog responsible for the pain-related status as those nerves? (iii) How can we explain that molecules involved in axon guidance, that is, the SLIT2/ROBO axis, and, more specifically, in repulsion (within developmental studies) could have such an important correlation with intratumoral nerve density?

From a clinical point of view, this report suggests that blockage of the ‘axon guidance' family and, more specifically, the SLIT2/ROBO pathway and its following signaling (as *β*-catenin/N-cadherin), may be a therapeutic approach to reduce PANR as well as consequent pathophysiologic impacts on PDA development and patient's fate (in the form of tumor recurrence and neuropathic pain). The role of the intratumoral microenvironment on PANR is a recent concept in PDA progression, and we look forward to future studies aimed at addressing the function(s) of other stromal molecules impacting on PANR as well as the translation of the pathway described here in other solid cancers associated with neural remodeling.

## Materials and Methods

### Human samples

Freshly frozen tissue samples of PDAs (*n*=4) were obtained from patients who had undergone surgery at the Department of Digestive Surgery. Before surgery, all patients had signed an informed consent form that had been approved by the local ethics committee (Agreement reference of CRO2 tissue collection: DC-2013-1857). One of the patients received preoperative chemotherapy for 2 months. Three patients underwent hemipancreaticoduodenectomy, and one underwent distal pancreatectomy. No distant metastases were revealed at initial diagnosis. Histological examination confirmed a diagnosis of PDA in all cases. Tumor staging was performed according to the International Union Against Cancer TNM System (the 6th edition).

### Mouse strains and tissue collection

Pdx1-cre;Ink4a/Arf^fl/fl^;LSL-Kras^G12D^ mice were obtained by crossing the following strains: Pdx1-cre, Kras^G12D^ and Ink4A^f/f^ mice kindly provided by Dr. D Melton (Harvard Stem Cell Institute, Cambridge, MA, USA), Dr. R Depinho (Dana-Farber Cancer Institute, Boston, MA, USA) and Dr T Jacks (David H Koch Institute for Integrative Cancer Research, Cambridge, MA, USA), respectively. Pieces of tumor pancreata were fixed in 4% (wt/vol) formaldehyde for further immunostaining analysis or prepared for RNA extraction. All animal care and experimental procedures were performed in agreement with the Animal Ethics Committee of Marseille.

### Xenografts

Patient-derived pancreatic tumor pieces (1 mm^3^) were embedded in Matrigel before subcutaneous implantation into the flank of adult male Swiss nude mice (Charles River Laboratories, Bois des Oncins, France) under isoflurane anesthesia (induction, 4% (vol/vol); maintenance, 1.5% (vol/vol)). Experimental procedures were performed using patient-derived pancreatic tumor pieces after agreement from the South Mediterranean Personal Protection Comity, under the reference 2011-A01439-32.

### Statistical analysis

Results are presented as average±S.D. All other comparisons (except Figure 6 and Supplementary Figure 5) were analyzed with the unpaired, two-sided, independent Student's *t*-test without equal variance assumption. Pearson's correlation analysis (SAS Software 9.2) was performed based on the comparison between Slit2 and *α*SMA expression, or nerve numbers.

### Laser microdissection and microarray analysis

Microdissection was performed in the microdissection laboratory of the PRIMACEN platform (University of Rouen, Mont-Saint-Aignan, France) in collaboration with Magalie Bénard. Frozen sections (20 *μ*m) were obtained from selected tissue samples. After a brief staining with hematoxylin and eosin, sections were dehydrated. A surface of ~2 × 10^6^ and 4 × 10^6 ^mm^2^ was microdissected for epithelial and stromal compartments, respectively, using the PALM system (P.A.L.M. Microlaser Technologies AG, Bernried, Germany). The microdissected material was immediately dissolved in a buffer containing *β*-mercaptoethanol and RNA carrier, and frozen before the RNA extraction was carried out with the RNAeasy Mini Kit (Qiagen, Courtaboeuf, France).

RNA extracted from microdissected stromal and tumoral cell samples from each patient was analyzed separately as follows: 15 *μ*g of total RNA was converted to cDNA using SuperScript Reverse Transcriptase (Invitrogen, Cergy Pontoise, France), and T7-oligo-d(T)^24^ (Genset, Paris, France) was used as a primer. Second-strand synthesis was performed using T4 DNA polymerase and *Escherichia coli* DNA ligase and then blunt ended by T4 polynucleotide kinase. cDNA was purified by phenol–chloroform extraction using phase lock gels (Brinkmann, Orléans, France). Then cDNAs were *in vitro* transcribed for 16 h at 37 °C using the IVT Labeling Kit (Affymetrix, Santa Clara, CA, USA) to produce biotinylated cRNA. Labeled cRNA was isolated using an RNeasy Mini Kit column (Qiagen). Purified cRNA was fragmented to 200-30 mer using a fragmentation buffer. The quality of total RNA, cDNA synthesis, cRNA amplification and cRNA fragmentation was monitored by capillary electrophoresis (Bioanalyzer 2100; Agilent Technologies, Massy, France). Fifteen micrograms of fragmented cRNA was hybridized for 16 h at 45 °C with constant rotation, using a human oligonucleotide array U133 Plus 2.0 (Genechip; Affymetrix). After hybridization, chips were processed using the Affymetrix GeneChip Fluidic Station 450 (protocol EukGE-WS2v5_450). Staining was carried out with streptavidin-conjugated phycoerythrin (SAPE; Molecular Probes, Eugene, OR, USA), followed by amplification with a biotinylated anti-streptavidin antibody (Vector Laboratories, Burlingame, CA, USA), and by a second round of SAPE. Chips were scanned using a GeneChip Scanner 3000 G7 (Affymetrix) enabled for high-resolution scanning. Images were extracted with GeneChip Operating Software (Affymetrix GCOS v.1.4). Quality control of microarray chips was performed using AffyQCReport software (Affymetrix).^34^

Background subtraction and normalization of probe set intensities was performed using the robust multiarray averaging (RMA) described by Irizarry *et al.*^35^ To identify differentially expressed genes between stromal and tumoral compartments in each tumor, gene expression intensity was compared using a moderated *t*-test and a Bayes smoothing approach developed for a low number of replicates.^36^ To correct for the effect of multiple testing, the false discovery rate (FDR) was estimated from *P*-values derived from the moderated *t*-test statistics.^37^ Analysis was performed using the affylmGUI Graphical User Interface for the limma microarray package^38^ and with Partek Genomics Suite (Partek Inc., St. Louis, MO, USA). We scored genes as differentially expressed if the fold change was >1.5 and *P*-value <0.05. Raw data were submitted to the GEO repository under the record number GSE50570.

### M&M bioinfo

Individual Affymetrix files were parsed and normalized with GCRMA using *Affy* and *GCRMA* packages with Bioconductor 3.0 (http://www.bioconductor.org/packages/release/bioc/html/chipseq.html). Differential analysis was performed with the SAM method (MeV, version 4.8.1) after application of a variance (top 20% of transcripts were kept). FDR was fixed to 6%, and 1001 genes were identified as differentially expressed in the stromal tissue compared with tumor tissue. Gene ontology (GO) enrichment was measured by a hypergeometric distribution and Bonferroni-corrected.

Heat map highlighting transcripts marked as ‘Axon Guidance' found to be significantly overexpressed in stromal tissues than in tumor tissues were generated with Gene-E from the Broad Institute (Cambridge, MA, USA). Represented values are normalized GCRMA expression values of individual genes. Each column is related to a single Affymetrix chip hybridized using the cRNA synthesized from individual stromal or tumor tissue. Red color represents higher gene expression values and blue represents lower expression.

### Cell isolation and primary CAF culture

Small pancreatic tissue blocks were obtained from the pancreas during surgery in patients with resectable pancreatic adenocarcinoma (see Xenograft methods section). The tumors were cut into small pieces of 1 mm^3^ using a razor blade. The tissue pieces were digested by collagenase IV (Sigma-Aldrich, Chesnes, France; C1889) for 30 min at 37 °C, washed with media, resuspended, passed through a cell strainer (100 *μ*M) and finally plated in a T75cm^2^ flask. Tissue blocks trapped in the cell strainer were seeded into 10 cm^2^ culture dishes to isolate more PSCs by the outgrowth method. Cells were cultured in DMEM/F12 medium (Invitrogen; 31330-038), 10% serum (Sigma-Aldrich; F7524), 2 mmol/l l-glutamine (Invitrogen; 25030-024), 1% antibiotic/antimycotic (Invitrogen; 15240-062) and 0.5% sodium pyruvate (Invitrogen; 11360-039) and used for passage 4–8. Primary CAF features were verified by IF for a positive *α*SMA staining and a negative CK19 staining.

### *In vitro* modeling of intratumoral microenvironment cell interactions

Panc1 and MiaPaca 2 human cell lines and the mouse pancreatic tumoral cell line PK4A were used for the epithelial compartment. Human primary fibroblasts or human CAFs, as well as murine macrophages (RAW 264.7), were used for the stromal compartment, and human Schwann cells (sNF 96.2) were used for the nerve cell compartment. All cell lines were obtained from the American Type Culture Collection (Manassas, VA, USA), except PK4A, human fibroblasts and CAFs, which are derived from primary cells lines obtained in our laboratory (see Materials and Methods). Cell lines were cultured in DMEM supplemented with 10% fetal bovine serum (Sigma-Aldrich; F7524) and 1% of antibiotic/antimycotic (Invitrogen; 15240-062). The combination of human and murine cell lines was important in our model as it permits to determine through QPCR analysis, by designing specific human or mouse primers, which gene expressions are modified in each cell type even when those cell types are cocultured.

For modeling of intratumoral microenvironment cell interactions, fibroblasts and macrophage were cultured (cell concentration is dependent on dishes size) in dishes coated with collagen 0.1% (Sigma-Aldrich) alone or together (1 : 1) for 24 h and then serum deprived for 12 h. Panc1, MiaPaca 2 and sNF were cultured in uncoated dishes for 24 h and then serum deprived for 12 h. Conditioned media (Md) from these cultures were used: Md F (fibroblasts alone); Md M (macrophages alone); Md FcoM (fibroblasts cocultured with macrophages); Md F+M (Md from fibroblasts alone mixed to Md from macrophages alone; 1 : 1); Md SNF (sNF 96.2 alone).

### Behaviors of primary sensory neurons

Pregnant rats at 15 days' gestation were killed by cervical dislocation (Wistar rats; Janvier, Le Genest, France) and the fetuses were removed from the uterus. DRG were collected, placed in ice-cold Leibovitz medium (L15; Invitrogen) and dissociated by trypsinization (trypsin, 0.05% Invitrogen) for 20 min at 37 °C. The reaction was stopped by the addition of DMEM containing 10% of fetal bovine serum in the presence of DNAase I (Roche, Meylan, France). The suspension was triturated with a 10 ml pipette and the cells were mechanically dissociated by several passages through the 21-gauge needle of a syringe. Cells were then centrifuged at 350 × *g* for 10 min at room temperature. The pellet of dissociated cells was resuspended in DMEM-Ham F12 (Invitrogen) containing 1% N2 (invitrogen), 1% penicillin–streptomycin (Invitrogen), 1% l-glutamine and 3 ng/ml NGF (PeproTech, Neuilly sur Seyne, France and Tebu, Perray, France). Cells were seeded on the basis of 15000 cells per well in a 96-well plate precoated with poly-l-lysine (Sigma-Aldrich). Plates were maintained at 37 °C in a humidified incubator with 95% air/5% CO_2_. Cells were cultured in classic culture medium or in a defined culture medium. On day 5, cells were fixed in a solution of 4% paraformaldehyde in PBS for 30 min at pH 7.4. After permeabilization with 0.01% saponin, cells were blocked for 2 h with PBS containing 10% goat serum and then incubated with primary *β*-tubulin antibody (Sigma-Aldrich). Revelation was carried out using Alexa Fluor 488 goat anti-mouse IgG (Molecular Probes). The nuclei of neurons were labeled by a fluorescent marker (Hoechst solution; Sigma-Aldrich). For each condition, 2 × 10 pictures per well were taken using AnalyzerTM 1000 (GE Healthcare, Aulnay, France) with x20 magnification. All images were taken under the same conditions and analyzed with Developer Software (GE Healthcare).

### Cell migration assay

Schwann cell migration was studied using the sNF96.2 cell line under various conditioned media on Boyden chambers. Culture inserts (BD, Le pont le Claix, France) with a porous membrane at the bottom (8 *μ* pores) were coated with a mix made of gelatin 0.1% and fibronectin 10 *μ*g/ml, and then were seeded with sNF96.2 (100 000 per insert) and placed into wells containing the conditioned media. Migration was performed for 4 h. After cleaning and briefly staining inserts with coomassie blue, migration was assessed by counting the number of colored cells in 10 high-power fields (magnification x20).

### QRT-PCR

RNA was extracted from cell lines using TRIzol (Invitrogen) according to the manufacturer's instructions. RNA was extracted from the pancreas of 8-week-old healthy mice (Kras^G12D^/Ink4A^f/f^) and PDA-earing mice (pdx1-cre/Kras^G12D^/Ink4A^f/f^) according to Chirgwin's procedure,^39^ and RNA quality control was determined using Agilent 2100 Bioanalyzer (Agilent Technologies, Santa Clara, CA, USA). cDNA was made from 1 *μ*g of total RNA using the ImProm-II Reverse Transcription System (Promega, Charbonnières, France) according to the manufacturer's instructions. QRT-PCR was performed using cDNA amplicons amplified with specific primers and the GoTaq qPCR Master Mix Kit (Promega) using a Mx3000P Stratagene system (Agilent Technologies). Relative expression was calculated as a ratio of the particular gene expression to a housekeeping gene expression (TBP).

### Immunofluorescence

Slides from frozen tissue samples or cultured cells were available for IF. Staining was performed using the following antibodies: *α*SMA mouse monoclonal (1 : 2, M-0851 (Dako, Les Ulis, France) or 1 : 200, A2547 (Sigma-Aldrich)), SLIT2 rabbit polyclonal (1 : 40, sc-28945; Santa Cruz Biotechnology, Heidelberg, Germany) and cytokeratin 19 mouse monoclonal (1 : 50, M-0888, Dako). Image quantification was carried out using Image J software (http://imagej.nih.gov/ij/).

### Immunohistochemistry

Slides from frozen human samples or formalin-fixed mouse samples were available for IHC. Staining was performed using the following antibodies: SLIT2 rabbit polyclonal (1 : 40, sc-28945; Santa Cruz Biotechnology), PGP9.5 rabbit polyclonal (1 : 800, ab-10404; Abcam, Cambridge, UK) and AML mouse monoclonal (1 : 200, A2547; Sigma-Aldrich).

### Reagents

For competition studies, blocking SLIT2 antibody (rabbit polyclonal, 1 *μ*g) and associated blocking peptide were obtained from Santa Cruz Biotechnology (sc-26601 and sc-26601P). Human recombinant SLIT2 (25 pg or 25 ng) was obtained from Abcam (ab82131). Each was added to conditioned medium during cell migration assays.

### siRNA transfection and reporter assay

Human fibroblasts were transiently transfected using SLIT2 siRNA (EHU068081; Sigma-Aldrich) or control siRNA (SIC001; Sigma-Aldrich) and ribocellin (BioCellChallenge, Toulon, France) according to the manufacturer's instructions. sNF96.2 cells were transiently transfected using ROBO1, ROBO2 or control siRNA (Origene, Rockville, MD, USA) and ribocellin (BioCellChallenge) according to the manufacturer's instructions. Conditioned media produced from cells were serum-deprived, and then collected for migration assays, immunoprecipitation or cytoplasmic/nuclear protein extraction. TCF/LEF reporter assay was performed according to the manufacturer's instructions (CCS-018L; SABiosciences, Courtaboeuf, France).

### Immunoprecipitation

sNF96.2 cells were incubated with conditioned media±siRNA for 30 min. Cell layers were washed in cold PBS and incubated for 10 min in lysis buffer. Cell lysates were cleared by centrifugation at 15000 × *g* for 15 min. Supernatants were incubated with N-cadherin antibody (rabbit polyclonal, 1 *μ*g, ab18203; Abcam) for 2 h at 4 °C before the addition of agarose beads. After 45 min of incubation with beads at 4 °C, the material was washed three times with lysis buffer. The immunoprecipitated and input material was eluted in the loading buffer, fractioned by SDS-PAGE, transferred to nitrocellulose membrane and immunoblotted with appropriate antibodies: N-cadherin (1 : 250, rabbit polyclonal, ab18203; Abcam) and *β*-catenin (1 : 2000, mouse monoclonal, 610153; BD Transduction Laboratories, Le Pont le Claix, France).

### Cytoplasmic and nuclear protein extraction

sNF96.2 cell were incubated with various conditioned media for 90 min. All steps were performed with the Nuclear Extract Kit (Active Motif, Carlsbad, CA, USA) according to the manufacturer's instructions. Nuclear extracts were resuspended in loading buffer, fractioned by SDS-PAGE, transferred to nitrocellulose membrane and immunoblotted with the appropriate antibody: *β*-catenin (mouse monoclonal, BD Transduction Laboratories, 1 : 2000) and Lamin A/C (rabbit polyclonal, Imgenex, 1/1000).

### Western blotting

For detection of SLIT2, total proteins were isolated from human PDA and healthy pancreas. Protein concentrations of the lysates were determined using the Bradford Protein Assay Reagent (Bio-Rad). Electrophoresis was carried out using XCell *Sure*Lock Mini-Cell (Invitrogen). The extracts (50 *μ*g/lane) were resolved by 3–7% NuPAGE Novex Tris-Acetate Mini Gels electrophoresis and electrotransferred onto an Immobilon polyvinylidene difluoride (PVDF) membrane (Immobilon-P^SQ^) using an electrophoretic transfer system (Invitrogen). PVDF membranes were divided into two parts according to the location of molecular weight markers to permit detection of both C-terminal protein Slit2 (about 55–60 kDa) and *β*-tubulin (49 kDa) by western blotting. The latter was used as an internal control. The membrane was blocked in freshly prepared PBS 1 × supplemented with 5% goat serum and 0.5% nonfat dry milk for 1 h at 37 °C. The membrane was then incubated overnight at 4 °C in a blocking buffer containing Slit2 (rabbit polyclonal antibody, 1 : 100; Santa Cruz Biotechnology) or *β*-tubulin antibody (mouse monoclonal antibody, 1 : 5000; Sigma-Aldrich), followed by three washes in TBST. Thereafter, the membrane was incubated with horseradish peroxidase-conjugated secondary antibody in TBS 1 × supplemented with 3% BSA (1 : 5000 dilution, goat anti-rabbit or goat anti-mouse IgG-HRP; Santa Cruz Biotechnology) for 1 h at 37 °C. The membranes were developed with an enhanced chemiluminescence substrate (Millipore, Fontenay sous Bois, France) and digitally scanned with Fusion Fx7 (Vilber Lourmat, Collegien, France).

## Abbreviations

*α*SMA: *α*-smooth muscle actin
CAF: cancer-associated fibroblast
IF: immunofluorescence
IHC: immunohistochemistry
PANR: PDA-associated neural remodeling
PDA: pancreatic ductal adenocarcinoma
SAM: significance analysis of microarray
