# Supplementary material for: Stromal SLIT2 impacts on pancreatic cancer-associated neural remodeling
Source: Cell Death Dis. 2015 Jan 15;6(1):e1592–. doi: 10.1038/cddis.2014.557 (PMC4669755; doi:10.1038/cddis.2014.557)
Supplement: Supplementary Figure Legends [file cddis2014557x1.doc]

**SI Figures**

**Supplemental Figure 1.** Determination of stromal and tumoral cell compartment transcriptomic signatures and characterization of the neurogenic factor family. (**A**) Schematic representation of Pancreatic Ductal Adenocarcinoma Associated Neural Remodeling. (**A**, **Left**) Neural Remodeling with infiltration of nerve fibers within Pancreatic Ductal Adenocarcinoma. (**Right**) Perineural Invasion representing the infiltration of tumoral cells within nerve fibers. (**B**) Graphical representation of technical procedure used to determine stromal and tumoral cell compartment transcriptomic signature from Human PDA samples (n=4). (**C**) Quality control of RNA obtained from microdissected area. RIN (RNA integrity Number) was measured by Agilent 2100 bioanalyzer. (**D**) Heat map highlighting transcripts marked as “Axon Guidance” found to be significantly over-expressed in stromal compared to tumor tissue. Each column is related to a single Affymetrix chip hybridized using the cRNA synthesized from individual stromal (S) or tumor (T). Red color represents higher gene expression values and blue represents lower expression.

**Supplemental Figure 2. “**Axon guidance” family expression *in vitro*. (**A**) Mouse mRNA level of some genes composing the “Axon guidance” family analyzed by QRT-PCR in mouse pancreatic tumoral cell line (PK4A, used as normalizer), mouse macrophages (M) and mouse macrophages cocultured with Human Fibroblasts. Light green, *P*<0.05 vs. PK4A; dark green, *P*<0.05 vs. M (n=3).

**Supplemental Figure 3.** Intra-tumoral microenvironment induces changes in primary sensory neurons and Schwann cell behaviors. (**A**) Representative images (all at 20X magnification, scale bar: 100 μm) from neuronal networks immunofluorescently labeled with β-tubulin as neuronal marker. (**B**) Human Schwann cell proliferation was measured by MTS assay after 24h or 48h.

**Supplemental Figure 4.** *In vitro* validation of SLIT2 and ROBO inhibition and localization. (**A**) Representative Western blot of SLIT2 on fibroblasts 72hrs after transfection with siRNA control (Si-Ctr) or targeting Slit2 (Si-Slit2). (**B**) Representative images (all at 20X magnification, scale bar: 100 μm) from nerve sections of Human PDA immunofluorescently labeled with Robo1 or GFAP, as Schwann cell marker. (**C and D**) Representative Western blot of ROBO1 (**C**) or ROBO2 (**D**) on SNF cells 72hrs after transfection with siRNA control (Si-Ctr) or targeting ROBO1 (Si-ROBO1) or targeting ROBO2 (si-ROBO2).

**Supplemental Figure 5.** Analysis of β-catenin nuclear translocation *in vitro* by immunofluorescence. (A) Nuclear translocation of β-catenin was analyzed by immunofluorescence with the same experimental protocol than Figure 5C. Representative images of β-catenin staining are shown. (B) Measure of relative luciferase unit representative of TCF/LEF activation in sNF 96.2 cells exposed to SNF (used as normalizer), F+M or FcoM media. Fibroblasts are transfected with control siRNA (si-Ctr) or SLIT2 siRNA (si-SLIT2) to obtain SLIT2-depleted media. (N=3). ***, *P*<0.001.

**Supplemental Figure 6.** Slit2 influences PDA associated neural remodeling within *in vivo* mice models. (**A**) Nerve counts (Intra-tumoral, Peri-tumoral and Total) on 14 pancreatic tumors from PDA bearing mice. Representative images (all at 20X magnification, scale bar: 100 μm) from intra-tumoral and peri-tumoral nerve labeled with PGP9.5 as marker. (**B**) Correlation between Slit2 expression and peri-tumoral nerve count (Left panel) or total nerve count (right panel) in pancreatic tumor samples from 14 PDA bearing mice. The Pearson correlation test showed a positive and significant correlation of 0.819 (*P*<0.01) and 0.918 (P<0.001), respectively. (**C**) Correlation between Slit2 expression and peri-tumoral nerve count (Left panel) or total nerve count (right panel) in 15 Human PDA xenograft samples. The Pearson correlation test showed a positive and significant correlation of 0.718 (*P*<0.01) and 0.802 (P<0.001) respectively.

**Supplemental Figure 7.** Summary diagram shows how intra-tumoral microenvironment impacts on PANR. Pancreatic Stellate cells/macrophages dialogue leads to Slit2 secretion by Pancreatic Stellate cells. Increased concentration of Slit2 enhances Neural Remodeling by increasing the infiltration of new nerve fibers within pancreatic tumors. Slit2-induced signaling in neurons and Schwann cells are mediated through its receptors Robo1/2 and consequent activation of N-cadherin/β-catenin pathway.
