# Supplementary material for: Stromal SLIT2 impacts on pancreatic cancer-associated neural remodeling
Source: Cell Death Dis. 2015 Jan 15;6(1):e1592–. doi: 10.1038/cddis.2014.557 (PMC4669755; doi:10.1038/cddis.2014.557)

A

B

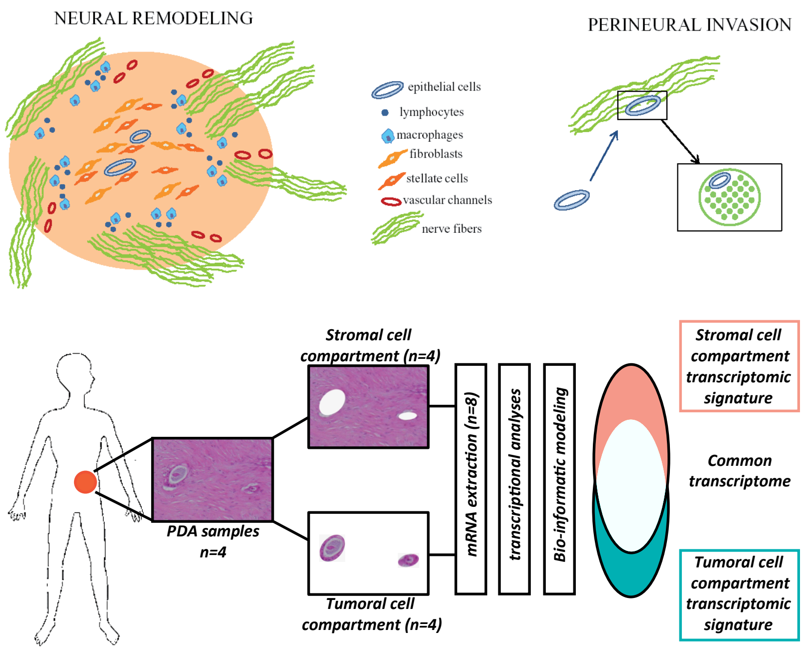

C

Compartment	Patient #	RIN
Stroma	1	6.2
Tumoral cell	1	7
Stroma	2	6.2
Tumoral cell	2	6.5
Stroma	3	6.6
Tumoral cell	3	6.6
Stroma	4	6.1
Tumoral cell	4	7.2

D

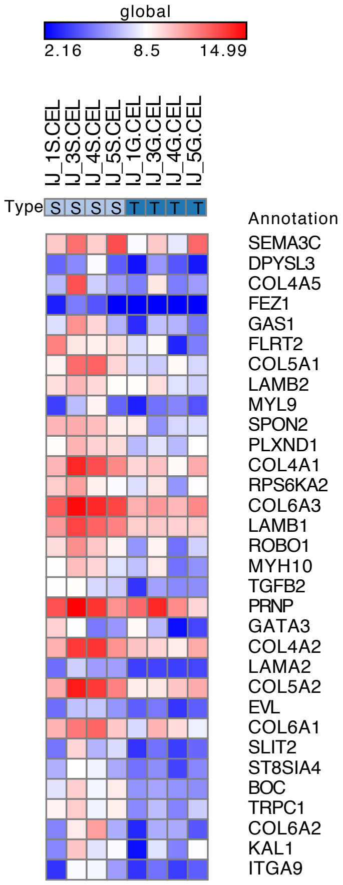

Supplement: Supplementary Figure 1 [file cddis2014557x2.pdf]
