# Supplementary material for: Stromal SLIT2 impacts on pancreatic cancer-associated neural remodeling
Source: Cell Death Dis. 2015 Jan 15;6(1):e1592–. doi: 10.1038/cddis.2014.557 (PMC4669755; doi:10.1038/cddis.2014.557)
Supplement: Supplementary Figure 2 [file cddis2014557x3.pdf]

A

Gene	Pk4A	M	FcoM
CNTN1	1	**5,83**	1,91
GAS1	1	0,4	1,1
ITGA9	1	1,5	0,9
L1CAM	1	**543**	0
DPYSL3	1	0	0
ROBO1	1	0,3	0,1
SLIT2	1	0	0
