# Supplementary figures and images for: Stromal SLIT2 impacts on pancreatic cancer-associated neural remodeling
Source: Cell Death Dis. 2015 Jan 15;6(1):e1592–. doi: 10.1038/cddis.2014.557 (PMC4669755; doi:10.1038/cddis.2014.557)

A

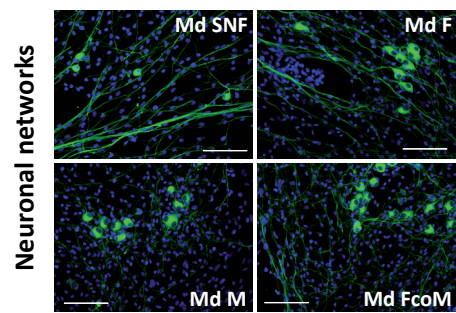

B

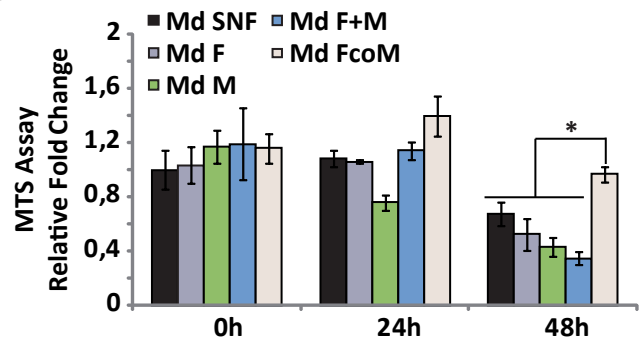

Supplement: Supplementary Figure 3 [file cddis2014557x4.pdf]

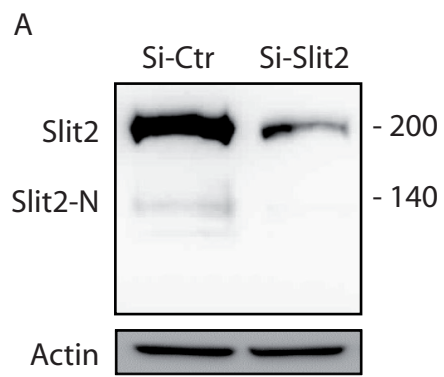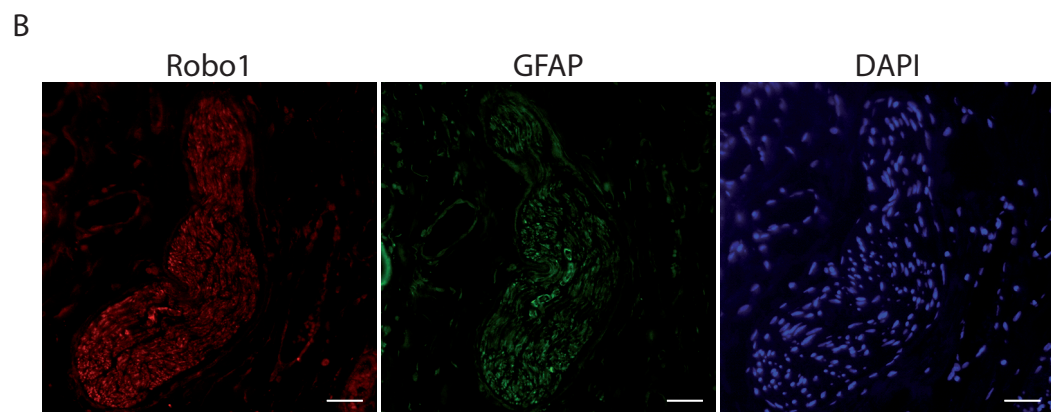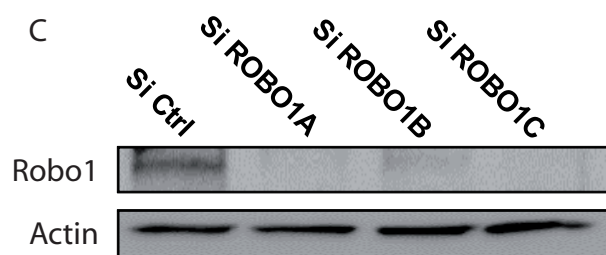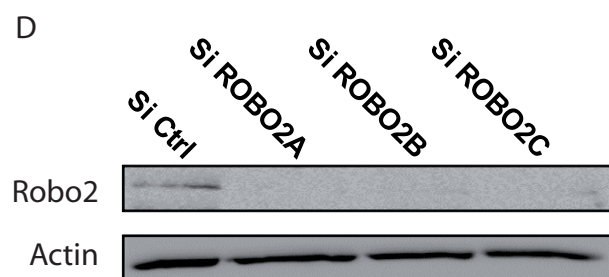

Supplement: Supplementary Figure 4 [file cddis2014557x5.pdf]

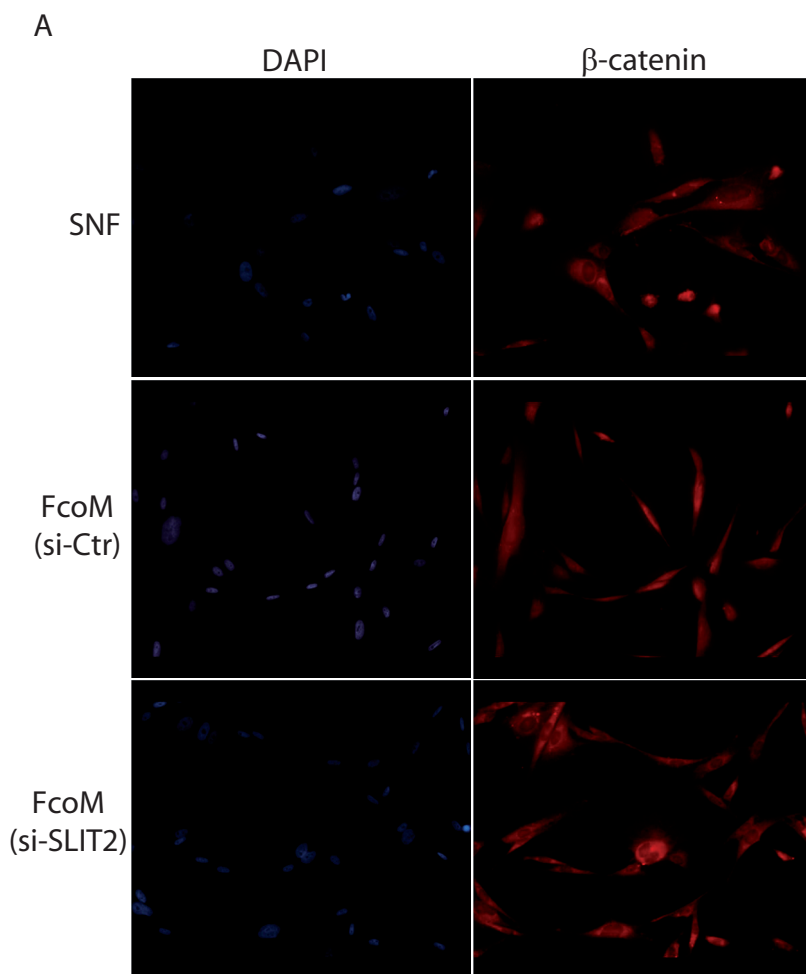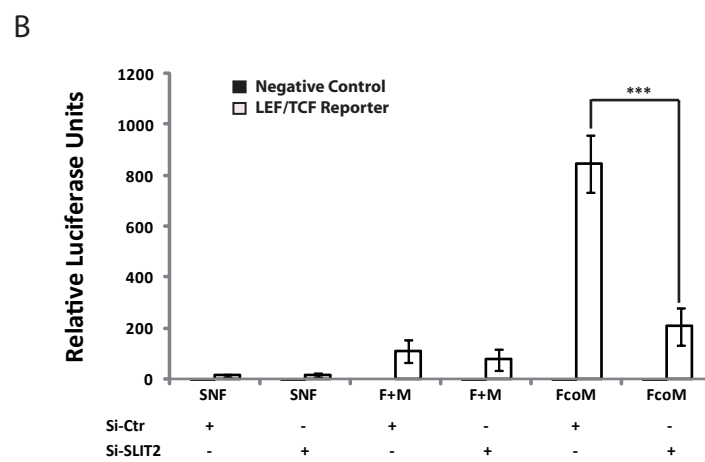

Supplement: Supplementary Figure 5 [file cddis2014557x6.pdf]

A

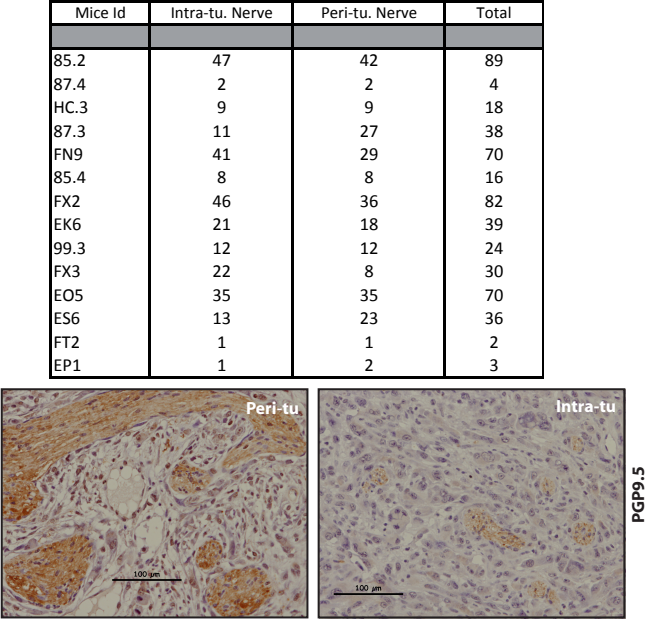

B

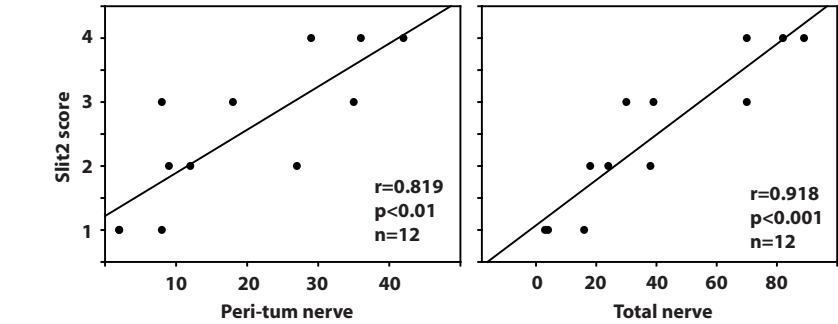

C

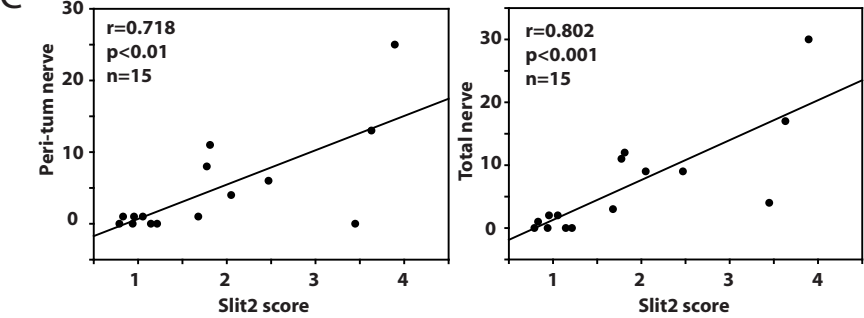

Supplement: Supplementary Figure 6 [file cddis2014557x7.pdf]

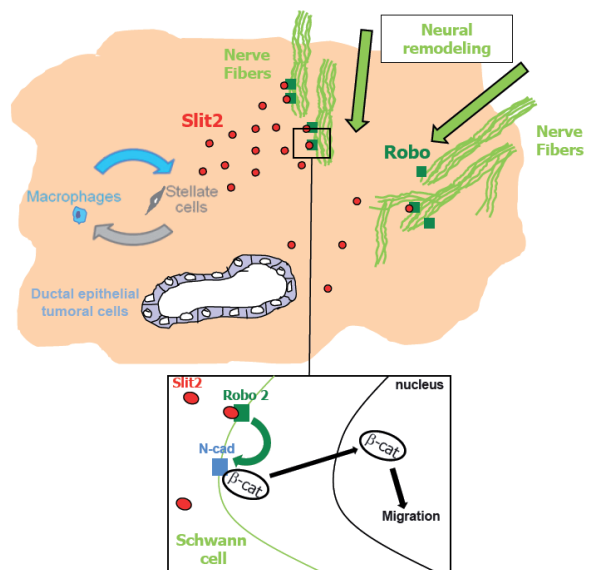

Supplement: Supplementary Figure 7 [file cddis2014557x8.pdf]
